# Mapping the Green Urban: A Comprehensive Review of Materials and Learning Methods for Green Infrastructure Mapping

**DOI:** 10.3390/s25020464

**Published:** 2025-01-15

**Authors:** Dino Dobrinić, Mario Miler, Damir Medak

**Affiliations:** Chair of Geoinformatics, Faculty of Geodesy, University of Zagreb, 10 000 Zagreb, Croatia; mario.miler@geof.unizg.hr (M.M.); damir.medak@geof.unizg.hr (D.M.)

**Keywords:** green infrastructure, machine learning, deep learning, urban ecosystem, green space, Scopus

## Abstract

Green infrastructure (GI) plays a crucial role in sustainable urban development, but effective mapping and analysis of such features requires a detailed understanding of the materials and state-of-the-art methods. This review presents the current landscape of green infrastructure mapping, focusing on the various sensors and image data, as well as the application of machine learning and deep learning techniques for classification or segmentation tasks. After finding articles with relevant keywords, the PRISMA (Preferred Reporting Items for Systematic Reviews and Meta-Analyzes) method was used as a general workflow, but some parts were automated (e.g., screening) by using natural language processing and large language models. In total, this review analyzed 55 papers that included keywords related to GI mapping and provided materials and learning methods (i.e., machine or deep learning) essential for effective green infrastructure mapping. A shift towards deep learning methods can be observed in the mapping of GIs as 33 articles use various deep learning methods, while 22 articles use machine learning methods. In addition, this article presents a novel methodology for automated verification methods, demonstrating their potential effectiveness and highlighting areas for improvement.

## 1. Introduction

**Figure 1:**
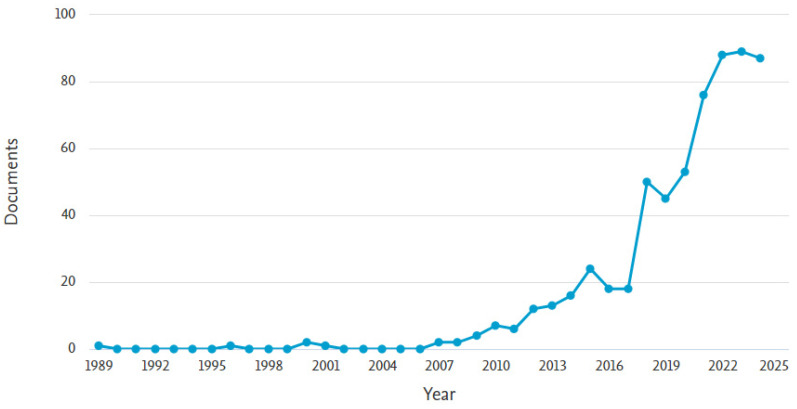
Number of papers with relevant keywords ‘green infrastructure mapping’ collected in the Scopus database.

Globally, urbanization has shown a rapid upward trend over the last seventy years, i.e., in 1950, 30% of the world’s population was urban and by 2050, 68% of the world’s population is expected to be urban [1]. Currently, 74% of Europe’s population live in cities, making it the third most urbanized region in the world after North America and Latin America [2]. With the majority of the population now living in urban areas, green infrastructure plays a key role in adapting to climate change [3,4], mitigating the heat island effect [5,6], and generally improving the quality of life of urban dwellers. Green infrastructure (GI) refers to an interconnected network of natural and semi-natural areas that are designed and managed to provide a wide range of environmental, social, and economic benefits. Unlike sustainable hard infrastructure, which may be considered “green” due to its energy efficiency or carbon reduction capabilities, GI is fundamentally rooted in living systems and ecological processes. It serves multiple purposes, including enhancing biodiversity, managing stormwater, reducing urban heat islands, and improving air quality. This approach promotes the conservation of functional ecosystems through strategic land-use planning and serves as a nature-based solution to mitigate environmental impacts, adapt to climate change, and build resilient communities [7,8]. However, as Honeck et al. [9] mention, the concept of GI has been defined and interpreted in different ways without a clear consensus on its components or the methods to identify and map it. Moreover, the authors of [10] did not find a single paper before 2005 that addresses green urban infrastructure and sustainable development. Thereafter, with the publication of the Millennium Ecosystem Assessment (March 2005) and after the United Nations Conference on Sustainable Development (2012), the number of published papers increased significantly [11], proving that GI is a topic that deserves further research and discussion (Figure 1).

Until now, the classification of GI spaces in urban areas has been difficult due to the heterogeneity of the landscape. However, with the availability of new data (e.g., high-resolution multispectral or radar imagery), the ability to recognize GI areas in cities has improved considerably [12]. For example, Figure 1 shows an increase in published papers after 2015 and 2017, which coincided with the launch of the Sentinel-2A and Sentinel-2B satellite, respectively. In addition to using the traditional four spectral bands for GI mapping (i.e., red, green, blue, and near-infrared), the recent SuperDove satellite by Planet Labs [13] enabled an additional four spectral bands with a spatial resolution of 3 m, which proved useful for distinguishing tree species in urban areas [14,15,16]. Despite the use of optical images with very high spatial resolution, the classification of urban trees in highly heterogeneous areas is challenging due to various factors such as shadow effects, variations in crown brightness, and background noise [17]. This suggests that the use or integration of non-spectral data, e.g., Light Detection and Ranging (LiDAR) or Synthetic Aperture Radar (SAR), can be utilized for the differentiation and classification of urban GI. In general, LiDAR data obtains 3D structural information by emitting laser pulses and measuring the time it takes for the pulses to return [18], providing information such as tree height, crown density, tree canopy structure, etc., while SAR is an active remote sensing technology that uses microwave radar signals [19]. By combining optical (vegetation type, condition) and SAR data (structural insights), a complete picture of urban green infrastructure can be obtained. For example, recent studies have integrated optical and LiDAR or SAR data for urban GI mapping [20,21,22,23]. In addition, various machine learning (ML) methods have been used in GI mapping research because they are simple and provide good classification results. Thus, ML methods such as K-nearest neighbor (KNN), random forest (RF), support vector machine (SVM), and extreme gradient boosting (XGB) are frequently used in GI mapping [24,25,26,27]. In recent years, convolutional neural networks (CNNs) have been shown to be very effective in object detection and image segmentation as they generate hierarchies that help determine low, medium, and high-level features [28]. These models are designed to process spatial data efficiently by using convolutional layers that preserve the spatial relationships between pixels, as opposed to, for example, pixel-based classification methods that classify each pixel individually [28,29].

Previous review papers have focused on specific aspects of green infrastructure studies, such as an economic study conducted within the framework of ecosystem services and GI [30], on the theoretical frameworks, and an interpretation of the role of GI for the ecology of cities [31]. Therefore, in order to bridge this research gap, this research will focus on a comprehensive review of the materials and machine learning methods that are essential for effective green infrastructure mapping. Furthermore, as the number of published papers on green infrastructure mapping is increasing (Figure 1), a novel method presented in [32] will be used to improve the scalability of the review process to keep pace with the rapidly growing research fields.

## 2. Methods

### Data Identification and Screening

The systematic literature search was conducted using Scopus, one of the largest abstract and citation databases indexing a wide range of international remote sensing journals and containing over 29,000 titles from more than 7000 publishers, ensuring comprehensive global research coverage [33]. The PRISMA (Preferred Reporting Items for Systematic Reviews and Meta-Analyzes) guidelines, a widely used method to ensure the reproducibility and transparency of systematic reviews, were followed in the selection of studies [34,35]. The search condition (Table 1) was used to detect papers that contained keywords related to green infrastructure or green spaces and to methods used in research (e.g., deep learning (DL) or machine learning). This search condition was limited to English-language journals and resulted in 314 potential papers.

**Figure 2:**
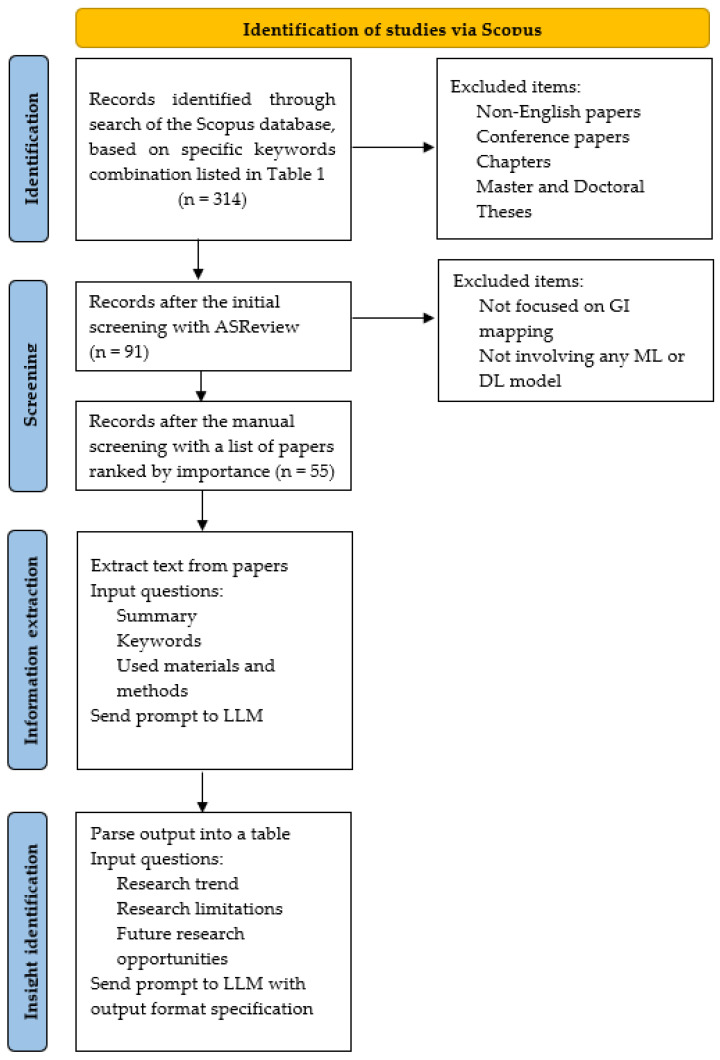
The overall workflow undertaken in this research, which can be divided into two major steps. The first step is similar to the screening process suggested by PRISMA guidelines, and the second step uses an LLM model for information extraction and insight identification.

After finding papers with relevant keywords, the PRISMA (Preferred Reporting Items for Systematic Reviews and Meta-Analyzes) method was used as a general workflow (Figure 2). However, some parts, e.g., screening, were performed using the ASReview software v1.0 developed by van der Schoot et al. [36]. This open-source ML tool enables the screening and systematic labeling of a large collection of textual data. First, relevant papers must be manually labeled by reading research abstracts. Then, active learning [37] is used to apply the feature extractor, train the model, and generate a list of papers ordered by importance (i.e., TF-IDF as the feature extraction technique that converts the text of each paper into numerical feature vectors and a Naïve Bayes as classifier that is trained on labeled data). At the end, papers are ranked based on the predicted probability of relevance, with higher probabilities indicating higher importance, and this ranking ensures that the most likely relevant papers appear at the top of the list. In addition, a snowballing technique [38] was used, similar to the research of Ito et al. [32], where a certain number of irrelevant papers must be set to stop labeling. This number was set to 10. As a result of this step, a total of 30% of the total papers were re-viewed and 59 papers were identified as relevant for this study. As the full text was not available for four papers, fifty-five papers were finally included in the review.

Following the proposed novel addition of the research from [32] to the PRISMA method for information extraction and insight identification, GPT-4 was used as the large language model (LLM) for text summarization as it has 97% and 100% accuracy in text summarization and information retrieval, respectively [39,40]. Here, Python was used as the programming language for text extraction from the papers to formulate prompts, which were then sent to the LLM. In this step (Figure 2), the prompts sent to the LLM were formatted to include information about the main findings of each paper and the materials and methods used in the research. The LLM output was then formatted into a table. When this step and the final table were exported (Figure 2), we manually checked 10% of the total papers to confirm that the LLM model had generated an appropriate output. In the final step (i.e., insight identification; Figure 2), the LLM was prompted to ask questions about research trends and limitations, as well as future research opportunities. The flowchart illustrating the review methodology is shown in Figure 2 and follows the PRISMA guidelines [34]. It also includes details of when each exclusion criterion was applied, in line with recent systematic review studies such as [10,11,41].

## 3. Results

### 3.1. Trends on Published Papers for Green Infrastructure Mapping

**Figure 3:**
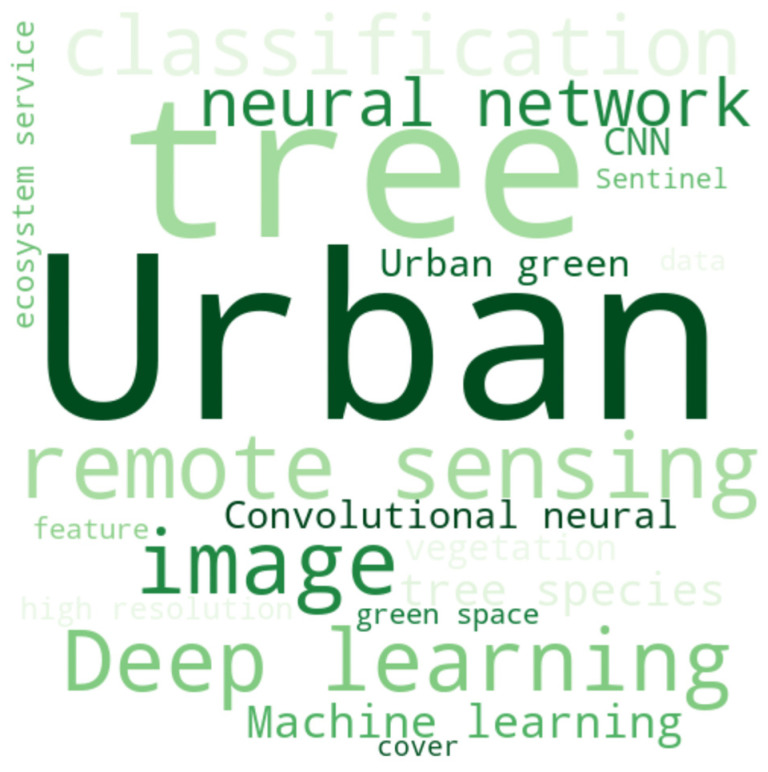
Word cloud showing the most frequently mentioned keywords in the papers that satisfied the query condition from Table 1.

As mentioned above, the European Commission defines green infrastructure as a “strategically planned network of natural and semi-natural areas with other environmental features designed and managed to deliver a wide range of ecosystem services” [42]. However, there is no agreement on the components of GI nor on the methods to identify and map them [43]. As a result, the concept of GI has been defined and linked in different ways [9], leading to the emergence of different terms and concepts to describe the same idea (e.g., green-prints, natural asset maps, ecological networks, green, blue, brown, and black corridors). Figure 3 therefore shows the most frequent keywords mentioned in the papers that met the search conditions in the Scopus database and the manual screening. The observed category, i.e., green infrastructure mapping, showed a high frequency of words such as ‘urban’, ‘image’, ‘tree’, ‘remote sensing’, and ‘deep learning’.

Because conference papers were excluded from our meta-analysis because they generally have a lower level of academic rigor than peer-reviewed articles, and because many of them are likely to be expanded into journal papers after being presented at conferences, Table 2 shows the journals that published at least two articles for the filtered 55 peer-reviewed papers.

**Figure 4:**
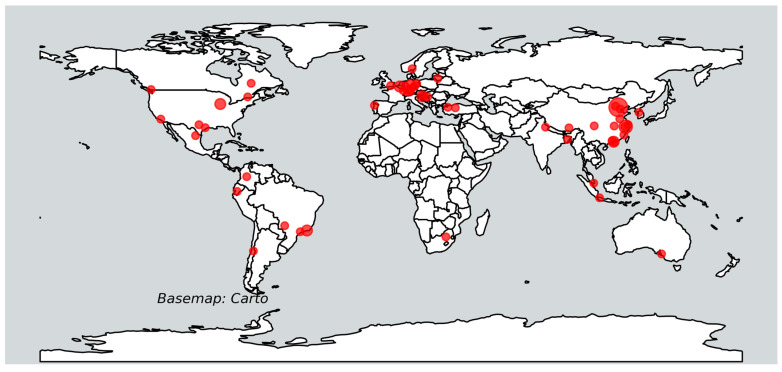
Distribution of the study areas used in the reviewed papers.

The research papers were conducted in different study areas in Asia, America, and Europe, and only one in Africa (Figure 4). The distribution shows that most of the research on GI mapping was conducted in Europe, particularly in countries such as Germany, Belgium, and Croatia. These studies covered a wide range of topics related to GI mapping in urban areas, from the comparison of different ML methods [44] to the classification of common tree species in urban environments [15]. Another hotspot that was used for research is in various locations in the city of Beijing. A total of six papers used the above-mentioned study area, and most of this research focused on deep learning methods [45,46].

### 3.2. Materials Used for Green Infrastructure Mapping

We currently live in the era of big data, where accurate and timely mapping of, for example, green infrastructure or urban green spaces can be important for various applications such as mitigating the urban heat island or promoting biodiversity [47,48]. Before the advent of airplanes and satellites, ground surveys were the primary method used for land-cover mapping [49]. Later, with the advent of aerial and satellite imagery, mapping became easier and cheaper as users gained an overview of the Earth’s surface [50]. Since the launch of Landsat-1 in 1972 [51], many users have started to create land use or land cover datasets at regional and national scales. However, in heterogeneous and complex landscapes, it is difficult to map information and patterns about green infrastructures because this class of optical sensors (e.g., MODIS, Landsat) is usually too coarse to accurately map GI [47,52]. With the introduction of new high-resolution space-based sensors such as WorldView, GeoEye, Pleiades, and SkySat with sub-meter spatial resolution, new opportunities for accurate GI mapping are emerging [25,53]. Further possibilities for GI mapping arise from sensors with different spectral and temporal resolutions [54]. The previously mentioned satellites use optical sensors but synthetic aperture radar (SAR) microwave sensors (e.g., Sentinel-1, ERS-1), which can provide imagery under all weather conditions, have also been used for GI mapping [55,56]. In addition, some satellite sensors acquire hyperspectral imagery, i.e., data with a very high spectral resolution. To characterize tree species in urban environments, Abbas et al. [57] and Mozgeris et al. [58] used hyperspectral images with 204 and 64 narrow bands, respectively. LiDAR [20,59] or street-view data [60,61] were also used in GI mapping to distinguish different tree species with different structures and components, or for information that is hidden from view from above.

According to the type of imagery used in GI mapping studies, we can divide the material into the following categories: optical, SAR, fusion, LiDAR, whether acquired through Airborne Laser Scanning (ALS) or Mobile Laser Scanning (MLS), aerial and hyperspectral images. Optical imagery was the most used for GI mapping with twenty papers, followed by aerial imagery, with nine papers. In contrast, SAR imagery was least frequently used independently for GI mapping [26,55,62], but mostly in combination with other data [22,23,56], such as optical imagery (e.g., Sentinel, Landsat). Another combination of data sources for GI mapping mainly includes optical imagery with aerial or SAR imagery [15,63], LiDAR data [20,59,64], and the integration of LiDAR and tree datasets [65]. In addition, LiDAR and hyperspectral datasets were mostly used for individual tree detection [57,58,66,67,68] or segmentation [69] in urban environments, or for the classification of urban vegetation patterns [70,71]. Aerial imagery with a spatial resolution of less than one meter was mainly used for deep learning-based GI mapping [72,73,74,75,76,77], while a smaller amount of research used them with machine learning methods [29,78,79].

**Figure 5:**
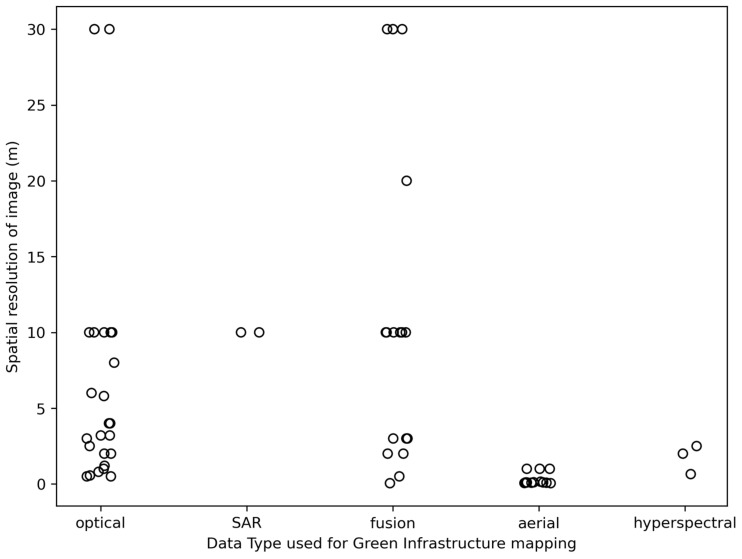
Distribution of image spatial resolution depending on the data type used for GI mapping.

As mentioned above, high-spatial resolution optical remote sensing (RS) imagery is still the most frequently used data source for GI mapping. The data sources for the case studies are mainly from RS satellite imagery such as Gaofen [45,46,80,81,82,83], Sentinel-2 [44,84,85,86,87], or the data from Digital Globe (e.g., WorldView, GeoEye, QuickBird) [25,28,47,53]. Some of the studies exclusively used RS imagery obtained from the PlanetScope [14,16], BJ-3N [88], Landsat [52], or SPOT6 [89] satellites. In addition, the spatial resolution of the data used determines the scope of the study. In general, high spatial resolution imagery is used for mapping individual trees, medium spatial resolution imagery is used for mapping GIs at the neighborhood or city level, and low spatial resolution imagery is used for the conservation of functional ecosystems at the regional and national level. The relationship between the spatial resolution of the imagery used in the case studies examined and the type of data used for GI mapping is shown in Figure 5.

There is another category used in recent studies for GI mapping, which is street view imagery, e.g., from Google, Baidu, and Mapillary [54]. The main advantages of these datasets are the affordable, seamless, and recurrent data collection for building tree inventories on a large scale [60,61]. Furthermore, Sun and Shi [90] and Pacheco-Prado et al. [91] acquired imagery for urban tree species detection using mobile smartphones, whereas Arevalo-Ramirez et al. [92] combined a public urban tree dataset with a large street-view urban tree dataset for the screening of urban trees. The only research that did not use remote sensing data was by Molina-Gomez et al. [93], who used a set of indicators to characterize the dimensions of sustainable development (e.g., environmental, social, economic, etc.) to classify the sustainability level of an urban area in Bogota.

### 3.3. Methods Used for Green Infrastructure Mapping

In recent years, the type of algorithms used to analyze and interpret spatial data has changed significantly in the field of green infrastructure mapping. In this review, deep learning methods were used for green infrastructure mapping in 33 papers, while machine learning algorithms were used in 22 papers. Traditionally, machine learning methods, including ensemble and tree-based algorithms such as Random Forest (RF), Support Vector Machine (SVM), and XGBoost, have been widely used for land cover classification, tree species identification, and urban-green-space mapping (Table 3). These algorithms are well suited for structured, tabular data and have demonstrated a high accuracy in spatial classification tasks due to their ability to process complex, high-dimensional datasets [62,64]. While these traditional machine learning algorithms have made a significant contribution to the field, recent advances in deep learning have paved the way for even more powerful techniques. As mentioned earlier, after the United Nations Conference on Sustainable Development in 2012, the number of published papers increased significantly, and this is one of the reasons why newly developed deep learning methods have been used for GI mapping. For example, an artificial neural network (ANN) was initially mainly used for urban tree species classification or tree canopy cover estimation [57,58,94]. ANN uses the backpropagation algorithm, a gradient-decent algorithm, and has one input layer, at least one hidden layer, and one output layer [95].

Deep learning algorithms such as convolutional neural networks (CNNs) and recurrent neural networks (RNNs) are particularly suitable for processing unstructured data such as satellite imagery and high-resolution spatial data (Table 3). These algorithms automatically learn complex patterns and spatial features, enabling more accurate and detailed mapping of green infrastructure [60,70]. In addition, advanced deep learning models based on the encoder–decoder (ED) CNN architecture, such as DeepLabv3+ or UNet (Table 3), are used for GI mapping as they can accurately segment complex features such as vegetation. The former model combines the advantages of the ED structure and the Atrous Spatial Pyramid Pooling (ASPP) module [96], while the latter skips the connections between ED parts, making it ideal for semantic segmentation tasks [28,59]. Also ResNet, a special type of Deep CNN that efficiently addresses the problem of vanishing gradients and degradation, has been used for tree species classification [15,91] or trunk segmentation [92].

In the context of green infrastructure mapping, YOLO (You Only Look Once) has also been used as an object detection algorithm. Choi et al. [61] used YOLO for the detection of tree species and the profile estimation of urban street trees present in Google Street View imagery. Furthermore, the PointNet++ deep learning algorithm and trainable superpixel segmentation were used for individual tree segmentation [69] or green roof classification [29]. The former research used tree point clouds as an input, while in the latter research an overall accuracy was not explicitly stated as the research reported the coverage of plant species cover. In addition, many papers introduced novel methods for mapping green infrastructure. These methods are mostly related to deep learning algorithms, i.e., the proposed methods are presented as improvements over existing methods and include applications such as urban green space segmentation [46], urban tree canopy mapping [53], tree species detection for urban greening [90], or the multi-scale segmentation of street trees [88]. The only research that tuned a ML algorithm was proposed by Zhou et al. [71], who investigated a hybrid approach of the fuzzy support vector machine tuned with a genetic algorithm for classifying urban vegetation patterns from hyperspectral imagery.

## 4. Discussion

The number of published papers on green infrastructure mapping is rapidly increasing every year (Figure 1), demonstrating its importance for sustainable urban planning and environmental management. This increasing demand has led to a notable shift from traditional machine learning techniques to more advanced deep learning approaches that offer higher accuracy and efficiency (Table 3). Machine learning methods, such as RF or SVM, offer simple and cost-effective approaches, making it accessible for smaller projects. Its limitations occur in scalability, where performance may degrade with increasing dataset size or geographic diversity, and a lower accuracy compared to DL methods. Alongside this methodological development, there is also a clear trend towards the use of higher resolution imagery—from low-resolution satellite data [47] to high-resolution satellite and drone imagery (Figure 5)—enabling more detailed and precise mapping of urban green spaces, vegetation, and ecological features.

Various materials, including LiDAR, satellite (RS) data, and street-view images, play critical roles in green infrastructure (GI) mapping. LiDAR data is highly effective for detailed vegetation and canopy analysis, satellite data offers broad spatial coverage and multi-temporal analysis, which is valuable for monitoring large-scale GI dynamics, and street-view imagery provides ground-level perspectives, enabling the identification of fine-scale features like street trees and small green spaces. However, some limitations in these GI mapping studies have also been reported. The limitations in [14] include the comparison with studies using LiDAR data in combination with very high-resolution satellite imagery, and the uncertainty related to the reference data [61,71,93] which are based on time-consuming field measurements. However, the use of reference data for the assessment may introduce potential biases or limitations, such as data imbalance or annotation errors [28,82,88], which could affect the validity of the results. Seasonal variations [57] may also affect the identification of certain tree species and the accuracy of structural measurements. While studies that used machine learning methods reported limitations such as limited phenological information and mixed signals from background and adjacent objects [16,45,83,92], papers that used deep learning methods mostly struggled with the problem of a limited sample size [72,78] and the computational infrastructure and time required to process the large-scale mapping of urban tree species [75,77,80,83]. Most studies using deep learning for green infrastructure mapping rely on large datasets of reference data to effectively train and validate models [73]. To increase the amount of training data, Pacheco-Prado et al. [91] recommend the inclusion of a wider range of data augmentation techniques [28]. Furthermore, Xu et al. [45] emphasize the importance of selecting optimal parameters for the loss function, as different datasets may require different threshold values [71]. Even though the studied work was conducted in different study sites (Figure 4), Zhang et al. [73] pointed out that extending the study to different locations and including a wider variety of tree species could provide more insights into the effectiveness of deep learning for tree species classification. In addition, some of the study sites were located in spectrally heterogeneous urban areas, which may pose a challenge to traditional classification methods [86,87]. Research that used LiDAR data for tree segmentation [69] has shown that over-segmentation can occur when extracting tree points, especially in areas with high tree density. The former research used an Optech Titan sensor operating in ALS mode with a typical vertical accuracy of 5–10 cm and horizontal accuracy of 1–5 cm and reported average point count per tree of 419, 912, and 1682 for the high, low, and medium density level, respectively. In addition, an incomplete representation of tree crowns due to gaps between LiDAR scan lines can affect the accuracy of the segmentation of individual trees. In some cases [80,84], misclassifications of shadows as green areas or water bodies occurred. Further analysis or validation for a wider range of shadow types and conditions may be necessary to verify the robustness of the algorithm. Also, some papers proposed novel methods for GI mapping (Table 3), and the authors pointed out that the generalizability of the models to a wider range of tree species or datasets from different regions or environments still needs to be explored [28,29,55,63,70,87,91]. Finally, some additional metrics or comparative analysis with other ML or DL methods are suggested to further validate the superiority of the proposed or tested methods [55]. Research could also explore adaptive frameworks that dynamically select relevant data layers based on task-specific needs, reducing computational costs while maintaining precision [97,98]. These directions hold promise for overcoming current limitations and paving the way for more efficient and reliable GI mapping solutions.

Future opportunities in green infrastructure mapping often reflect the era in which the research was conducted, determined by the technologies and materials available at the time. Early studies emphasized the advances in machine learning and the potential of medium to high resolution satellite imagery. As future research opportunities in the field of GI mapping, suggestions were made to improve classification accuracy by refining the ML algorithms used in the research [16,29,87,89] and especially to integrate data from multiple sources, such as LiDAR, hyperspectral, or aerial imagery [57,64,75]. As mentioned in the research from Degerickx et al. [20], ALS LiDAR data was found to be the most valuable dataset overall, while fusion with hyperspectral data was essential for mapping the most detailed classes. Furthermore, beside ALS LiDAR data, remarkable results were achieved in [68], where the RIEGL MLS system, with a mean point density of 6000 points per square meter, was used for the rapid acquisition of dense point clouds that can be used to extract vegetation. Also, Cheng et al. [46] suggested the integration of additional features such as texture features and elevation information (e.g., digital elevation model) [26] to improve the accuracy of urban green space segmentation, whereas Griffiths et al. [56] noted the redundancy of different input bands in the dataset and the need for further investigation to optimize the selection of input features. The study by Dobrinić et al. [99] evaluated different methods of feature selection, reducing the original dataset from 114 to 34 input features. More recent work utilizing deep learning methods for GI mapping has mainly focused on expanding datasets [91] and improving network architectures to overcome challenges such as complex backgrounds [67,76,92]. The former goal is necessary to avoid overfitting and to achieve good generalization to new unseen data, whereas the latter can affect the performance of segmentation algorithms, especially in areas with dense buildings [88]. Overall, deep learning models like CNNs have been used to achieve high accuracy in segmenting tree canopies from high-resolution satellite or aerial imagery, and once trained, models can rapidly analyze large areas, saving time and resources compared to manual interpretation. However, as mentioned in [76], training models require access to high-performance computing resources, which can be expensive, and the object-based approach, hyperparameter optimization, and model selection can be technically challenging for non-experts.

Finally, some novel parts that were used in this report should be highlighted (Figure 2). Screening time was considerably shortened using the ASReview software developed by van der Schoot et al. [36]. This open-source ML tool enables the screening and systematic labeling of a large collection of textual data. Systematic reviews are usually time- and resource-intensive, requiring a median of five researchers and 41 weeks to submit to a journal [100]. Nowadays, state-of-the-art strategies are being developed to automate systematic literature reviews [101]. After paper identification and screening of the articles, an LLM (i.e., OpenAI’s GPT-4 model) was used to extract information and identify findings. As mentioned by Ito et al. [32], when using an LLM one needs to pay special attention to hallucinations (i.e., generation of non-factual information), and they suggest an extensive examination of the method used for the automation of a systematic review in order to make a rigorous validation of the accuracy and quality of the outputs. Therefore, we manually checked 10% of the total papers to confirm that the LLM model generated a reasonable output.

## 5. Conclusions

Green infrastructure can be defined as a strategically planned network of natural and semi-natural spaces and other environmental features that provide ecosystem services, promote biodiversity, and support sustainable development. By integrating GI into urban planning, cities can achieve sustainable development while fostering resilience to climate change and other environmental challenges. The concept of green infrastructure has been defined and interpreted in different ways, with no clear consensus on its components or the methods to identify and map them. Therefore, in our study, we reviewed 55 papers that contained keywords related to green infrastructure or green spaces and to methods (e.g., deep learning or machine learning). In addition to analyzing the methods used for GI mapping, we also summarized the materials used in the research (e.g., satellite, LiDAR, or aerial imagery). Recently, there has been a shift towards the use of deep learning as opposed to machine learning methods. Deep learning methods have outperformed traditional machine learning approaches in cases where sufficient ground truth data were available. However, it is important to note that a higher resolution does not always guarantee better results, due to a trade-off between resolution and the specific task of green infrastructure identification. High spatial resolutions can increase data complexity and processing time without necessarily enhancing the accuracy for certain tasks.

To summarize, the results of our research provide a comprehensive overview of the materials and methods commonly employed in green infrastructure mapping, serving as a basis for future studies to refine and expand these approaches. However, there are some limitations to the mapping of urban green spaces. These include the generalizability of the models to a wider range of tree species or datasets from different regions or environments, as well as the uncertainty of the reference data. In addition, this paper presents a novel methodology for automated review techniques, demonstrating their potential effectiveness and highlighting areas for improvement.

## Figures and Tables

**Table 1:** Search condition used to detect relevant papers in the Scopus database made on 1 August 2024.

TITLE-ABS-KEY (
(“green infrastructure” OR “urban green infrastructure” OR “urban green space*” OR “urban ecosystem*”) AND (“machine learning” OR “deep learning” OR “neural networks” OR “convolutional neural network*” OR “CNN”) ) AND (LIMIT-TO (DOCTYPE, “ar”)) AND (LIMIT-TO (LANGUAGE, “English”))

**Table 2:** Number of the most relevant papers concerning green infrastructure mapping published in the international journals.

Name of Journal	#
Remote Sensing	9
Urban Forestry and Urban Greening	9
ISPRS Journal of Photogrammetry and Remote Sensing	5
Forests	4
Sustainability	3
Data	2

**Table 3:** Summary of machine learning or deep learning methods used for green infrastructure mapping.

**Methods**	**Application**	**Metrics**	**References**
ANN	Classification of urban tree species	OA 63–96%	[57,58,78,85]
	Modeling/estimation of tree canopy cover	OA 89–95%, R^2^ 0.95	[47,87,94]
	Forecast the sustainability levels of an urban ecosystem	OA 96%	[93]
Machine Learning Algorithms	Tree species mapping (SVM, RF, XGBoost)	OA 73.13–97%	[14,16,64,86]
	Estimate green volume (GV) in urban areas	R^2^ 0.775; RMSE 2.68	[22]
	Green infrastructure mapping	OA 78.32–97%	[23,26,44]
	Urban growth mapping	OA 83.5% to 84.7%; 94.17%	[52,56]
	Classifying blue-green-gray infrastructure	OA 84.9–94.0%	[79]
	Classification of urban green space types or green roofs	OA 89.93%; 98.26%	[63,89]
	Fill data gaps in existing tree inventories	From 19% to 54%	[65]
	Generation of detailed ecosystem service maps	OA 79–94%	[20,25]
DeepLabv3+	Tree species mapping in (tropical) urban areas	OA 92%; F1 87.6%	[72,84]
UNet	Urban tree canopy mapping	OA 96.40–99.14%	[59,74,76]
	Urban water extraction	IoU 93.06%	[55]
	Mapping of urban green spaces	OA 97.0–97.34%	[28,83]
	Urban tree detection from point clouds (ALS)	Precision 82.9%	[66]
(R) CNN	Urban feature extraction	OA 95.94–96.32%	[70,77]
	Detection of individual trees in urban areas	F1 0.88–0.95; ME 4–6 m	[60,67,75]
	Urban water bodies extraction	OA 99.14%	[80]
ResNet	Tree species classification in urban areas	OA 88.0–92.6%	[15,73,91]
	Trunk segmentation and tree genus classification	IoU 88%	[92]
YOLO, Point Cloud Architectures	Tree species detection and profile estimation of urban street trees	mAP 0.564; NRMSE 0.24	[61]
	Determination of green view index (GVI); MLS	GVI > 15%	[68]
	Individual tree segmentation (ALS)	OA 96%; F1 92.8%	[69]
	Green roofs classification to estimate plant species cover	NA	[29]
Novel methods	Urban green space segmentation	OA 92.90%; MIOU 74.64%	[46,82]
	Urban tree canopy mapping	OA 95.76%	[53]
	Multi-scale segmentation of street trees	OA 96.14%	[88]
	Classification of urban green spaces	OA 93.24–98.30%	[45,81]
	Classification of urban vegetation patterns	OA 71.2%; K 0.69	[71]
	Detection of urban greening tree species	OA 84.25%	[90]

## Data Availability

Data are contained within the article.
